# Revolutionizing Breast Cancer Diagnosis: A Concatenated Precision through Transfer Learning in Histopathological Data Analysis

**DOI:** 10.3390/diagnostics14040422

**Published:** 2024-02-14

**Authors:** Dhayanithi Jaganathan, Sathiyabhama Balasubramaniam, Vidhushavarshini Sureshkumar, Seshathiri Dhanasekaran

**Affiliations:** 1 Department of Computer Science and Engineering, Sona College of Technology, Salem 636005, India; sathiyabhama@sonatech.ac.in; 2 Department of Computer Science and Engineering, Faculty of Engineering and Technology, SRM Institute of Science and Technology, Vadapalani Campus, Chennai 600026, India; vidhushasuresh@gmail.com; 3 Department of Computer Science, UiT the Arctic University of Norway, 9037 Tromsø, Norway

**Keywords:** deep learning, histopathology images, breast cancer diagnosis

## Abstract

Breast cancer remains a significant global public health concern, emphasizing the critical role of accurate histopathological analysis in diagnosis and treatment planning. In recent years, the advent of deep learning techniques has showcased notable potential in elevating the precision and efficiency of histopathological data analysis. The proposed work introduces a novel approach that harnesses the power of Transfer Learning to capitalize on knowledge gleaned from pre-trained models, adapting it to the nuanced landscape of breast cancer histopathology. Our proposed model, a Transfer Learning-based concatenated model, exhibits substantial performance enhancements compared to traditional methodologies. Leveraging well-established pretrained models such as VGG-16, MobileNetV2, ResNet50, and DenseNet121—each Convolutional Neural Network architecture designed for classification tasks—this study meticulously tunes hyperparameters to optimize model performance. The implementation of a concatenated classification model is systematically benchmarked against individual classifiers on histopathological data. Remarkably, our concatenated model achieves an impressive training accuracy of 98%. The outcomes of our experiments underscore the efficacy of this four-level concatenated model in advancing the accuracy of breast cancer histopathological data analysis. By synergizing the strengths of deep learning and transfer learning, our approach holds the potential to augment the diagnostic capabilities of pathologists, thereby contributing to more informed and personalized treatment planning for individuals diagnosed with breast cancer. This research heralds a promising stride toward leveraging cutting-edge technology to refine the understanding and management of breast cancer, marking a significant advancement in the intersection of artificial intelligence and healthcare.

## 1. Introduction

### 1.1. About Cancer Statistics

**Figure 1:**
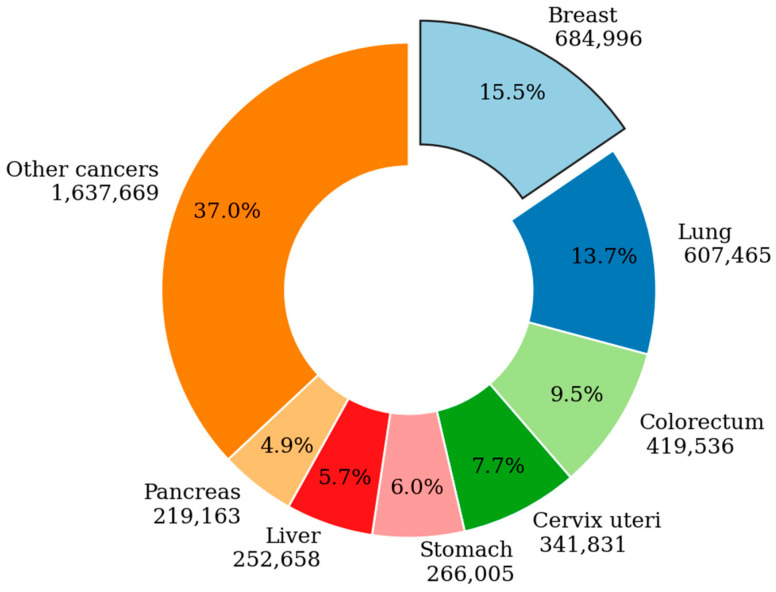
Globoscan cancer mortality 2023.

In 2023, the International Agency for Research on Cancer, through GLOBOCAN estimates, reported a substantial global cancer burden [1]. Worldwide, there were around 19.3 million new cases of cancer (except from nonmelanoma skin cancer) and almost 10.0 million deaths from cancer. Breast cancer is projected to cause 15.5% fatalities, followed by lung cancer with 13.7% deaths. Colorectal cancer is expected to account for 9.5% deaths while cervical cancer could result in 7.7% fatalities. Stomach cancer is predicted to cause 6.0% deaths, liver cancer 5.7% deaths, and pancreatic cancer 4.9% deaths. Additionally, other types of cancers are projected to lead to 37.0% deaths. Notably, female breast cancer surpassed lung cancer as the most frequently diagnosed cancer, with 2.6 million new cases. The incidence of cancer was 2 to 3 times higher in transitioned countries compared to transitioning ones for both sexes. However, mortality rates showed less disparity, with less than a 2-fold difference for men and minimal variation for women. Notably, death rates for female breast cancer were considerably higher in transitioning countries (15.0 and 12.4 per 100,000, respectively) compared to transitioned countries (12.8 and 5.2 per 100,000, respectively), and the details of cancer mortality are depicted in Figure 1.

Projections for 2040 indicate a significant increase in the global cancer burden, reaching 28.4 million cases, marking a 47% rise from 2020. Transitioning countries are expected to experience a larger surge (64% to 95%) compared to transitioned countries (32% to 56%). This escalation is attributed to demographic changes, potentially exacerbated by increased risk factors associated with globalization and economic growth. Addressing this challenge necessitates concerted efforts to establish sustainable infrastructures for disseminating cancer prevention measures and providing cancer care, particularly in transitioning countries, emphasizing the critical role of global cooperation in cancer control [1].

### 1.2. Breast Cancer and Its Treatments

One of the most prevalent malignancies in women globally, breast cancer is a malignant tumor that develops in the breast cells. For treatment to be successful, early detection is essential. A painless lump or thickening in the breast or underarm, changes in breast size, shape, or skin texture, an inexplicable nipple discharge, and skin redness or dimpling that resembles an orange peel are common signs of breast cancer. These symptoms may have benign causes as well as being suggestive of breast cancer. As a result, any enduring or strange modification to the breasts must be examined right away by a medical expert [2].

Treatment for breast cancer varies depending on the stage, type, and characteristics of the tumor. The primary treatment modalities include surgery (lumpectomy or mastectomy), radiation therapy, chemotherapy, targeted therapy (such as Herceptin for HER2-positive cancers), and hormone therapy (for hormone receptor-positive cancers). Often, a combination of these treatments is used to effectively combat the cancer. Additionally, advancements in personalized medicine have led to more tailored treatments based on the specific genetic and molecular characteristics of the tumor. Early-stage breast cancer typically has a better prognosis, but advancements in treatment have also improved outcomes for advanced-stage cancers. Regular breast self-exams, clinical breast exams, and mammograms are essential for early detection and increased chances of successful treatment [1].

### 1.3. Artificial Intelligence Based Computer Aided Diagnosis

Due to the rapid development of Artificial Intelligence (AI) techniques and Computer Aided Diagnosis (CAD) models, breast cancer histopathological data analysis made significant strides. These models leverage the power of AI to assist pathologists in detecting, diagnosing, and characterizing breast cancer [3]. AI and CAD models can automatically detect cancerous regions and anomalies within histopathological images. They can identify subtle features and patterns that may be challenging for human pathologists to detect, leading to earlier and more accurate cancer detection. These models serve as valuable tools for pathologists, aiding in the screening and review of large volumes of histopathological slides. Pathologists can benefit from AI-generated annotations and highlights, allowing them to focus their expertise on critical areas.

AI enables quantitative analysis of histopathological images [3]. It can measure various characteristics, such as tumor size, shape, density, and the presence of specific biomarkers. This quantitative data aids in diagnosis, prognosis, and treatment planning. AI models can classify breast cancer into different subtypes based on histological features. For example, they can distinguish between invasive ductal carcinoma, invasive lobular carcinoma, and other subtypes which guide treatment decisions. AI-driven prognostic models use histopathological data to predict patient outcomes, including survival rates and the likelihood of cancer recurrence and this information assists clinicians in tailoring treatment plans. AI systems can provide treatment recommendations by analyzing histopathological data alongside other patient information, such as genetic profiles and clinical history which support the development of personalized treatment strategies [3]. 

The authors have tested various machine learning (ML) models and examined the effects of using and not using the Synthetic Minority Oversampling TEchnique (SMOTE). Metrics including Area under the Curve (AUC), accuracy, precision, and recall were used to compare and completely assess the models. The results showed that the stacking ensemble model performed better than other models when combined with SMOTE and a 10-fold cross-validation strategy. Notable performance values were attained by this configuration: 90.9% accuracy, 96.7% precision, 87.6% recall, and 96.1% AUC [4].

CAD models can analyze histopathological slides much faster than humans, leading to quicker diagnoses and treatment decisions. This is especially crucial in cases where rapid intervention is required. AI can help maintain quality control by flagging ambiguous or potentially erroneous diagnoses for further review by pathologists. This reduces the risk of misdiagnoses. AI systems can integrate histopathological data with other types of medical data, such as radiological images and patient records, to provide a comprehensive view of the patient’s condition. AI models can continuously learn and adapt to evolving data and medical knowledge. This ensures that the system’s performance improves over time, benefiting from the accumulation of more data and experience.

The advent of digital pathology has ushered in a transformative era, revolutionizing the entire diagnostic workflow in pathology [5]. This technological shift has facilitated the application and advancement of AI models in pathology, leading to the generation of extensive pathological big data and the implementation of telepathology. AI algorithms, encompassing ML and Deep Learning (DL), now play pivotal roles in tasks such as detecting, segmenting, registering, processing, and classifying digitized pathological images. These pathological AI algorithms find valuable applications in diagnostic screening, conducting morphometric analyses of biomarkers, uncovering new prognostic and therapeutic insights within pathological images, and enhancing diagnostic efficiency. This review delved into the advantages and prospects offered by digital pathology, explored AI-based approaches, examined their diverse applications in pathology, and addressed the crucial considerations and challenges entailed in the development of pathological AI models [5].

The remarkable advancements in the accuracy of AI in the medical field have been notable. However, there is a limited understanding of the actual impact these algorithms might have on the decisions made by pathologists in their practical work. The authors of [6] aimed to investigate the dependence of pathologists on AI and explore how providing information about AI influences this reliance. The study employed an online survey design involving 116 pathologists and pathology students under three distinct conditions. The Gleason grade for a set of twelve prostate biopsies was to be evaluated by the participants in three different ways: (1) without AI recommendations, (2) with AI recommendations, and (3) with AI recommendations combined with details about the algorithm itself, such as its accuracy rate and decision-making procedure. The findings showed that participant responses with AI recommendations supported were substantially more accurate than assessments without AI (92% vs. 87%, odds ratio 13.30, *p*  < 0.01). When compared to AI without information, the algorithm did not significantly alter anything. It was discovered that the use of AI was correlated with broad opinions about its value but not with evaluations of the AI tool provided. Furthermore, when AI advices were given, judgments were taken more quickly [6].

In many cases surgery and biopsy procedures may not be required by having their breast cancer identified. The performance of deep learning (DL) systems used for medical image processing has significantly improved because of recent advances in the field. The goal of extracting significant features from histopathologic BC images is achieved by the extensive application of DL models. Both the classification performance and the process automation have benefited from this. Both hybrid models of DL-based approaches and convolutional neural networks (CNNs) have shown outstanding performance recently. Three distinct CNN model types—a simple CNN model (1-CNN), a fusion CNN model (2-CNN), and a three CNN model (3-CNN)—are offered in this study. The results show that the methods based on the 3-CNN algorithm worked the best in terms of the f1-Score (89.90%), recall (89.90%), accuracy (90.10%), and precision (89.80%). The accuracy of classifying breast cancer has significantly increased because of the use of CNN-based techniques [7].

Breast cancer histopathological datasets consist of thousands of microscopic images of breast tissue samples that have been labeled by pathologists to indicate the presence of cancerous cells or tissue characteristics associated with breast cancer [5]. These datasets are invaluable for training DL models to aid in the early detection of breast cancer, ultimately increasing patient survivability. DL models, especially CNNs, can be trained on histopathological images to recognize subtle cellular and tissue-level changes indicative of breast cancer [6]. These models learn to detect cancerous cells, tissue structures, and abnormalities in breast tissue samples with a high degree of accuracy. Early detection through such automated systems can lead to early intervention and treatment, significantly improving patient outcomes. 

DL algorithms can achieve high sensitivity (the ability to correctly identify true positive cases) and specificity (the ability to correctly identify true negative cases) in breast cancer detection. This reduces the likelihood of false negatives (missed cancer cases) and false positives (incorrectly flagged as cancer), leading to more reliable diagnoses and reducing unnecessary anxiety and procedures for patients. Automation of the screening process with DL models can help pathologists prioritize cases that require their expertise. By pre-screening and flagging potentially cancerous regions within histopathological images, pathologists can focus their attention on the most critical areas, making the diagnostic process more efficient [5]. DL models provide consistent and reproducible results. Unlike human pathologists, models do not experience fatigue or variability in their assessments. This consistency can lead to more reliable diagnoses and reduce the chances of missing cancers due to human error.

To assist in identifying specific molecular and genetic characteristics of tumors and helping to tailor treatment plans for individual patients, DL models play a vital role. This is referred to as precision medicine, and ensures that patients receive therapies that are most likely to be effective for their specific cancer subtype. Analyzing breast cancer histopathological datasets with DL can reveal patterns and insights that may not be apparent to the human eye. This can lead to a better understanding of the disease’s progression, subtypes, and treatment responses, ultimately contributing to ongoing research efforts. 

Despite these significant advantages, it’s important to note that AI and CAD models are not meant to replace pathologists but rather to enhance their capabilities. Human expertise remains essential in interpreting results, making final diagnoses, and considering the broader clinical context. AI and CAD models have emerged as valuable tools in breast cancer histopathological data analysis [5]. They offer increased accuracy, efficiency, and quantitative insights, ultimately leading to earlier detection, better treatment decisions, and improved patient outcomes in the fight against breast cancer.

Early detection and accurate diagnosis enabled by DL models can lead to timely intervention, which is crucial in cancer treatment. If breast cancer is diagnosed earlier, then more treatment options are available, and the better the chances of achieving a favorable outcome. This, in turn, increases the survivability of breast cancer patients. Breast cancer histopathological datasets analysis using DL models has the potential to revolutionize the early detection and management of cancer [8]. These AI-powered systems can enhance the accuracy, speed, and consistency of breast cancer diagnosis, leading to more timely interventions, personalized treatments, and ultimately, improved survival rates for patients. Histopathological analysis of breast tissue slides is vital for diagnosing and categorizing breast cancer. However, manual evaluation by pathologists can be time-consuming and subject to interobserver variability. DL has emerged as a promising tool for automating this process, with transfer learning being a powerful approach. Transfer learning leverages pre-trained models on large datasets and fine-tune them for specific tasks.

## 2. Literature Review

Mammograms, Ultrasound and Histopathology are the most common medical imaging modalities for breast cancer screening [9]. DL models are applied to detect and classify lesions, microcalcifications, and masses in mammograms and analyze ultrasound images for abnormalities. DL techniques can be applied to digitized histopathology slides to identify cancerous regions, grade tumors, and predict patient outcomes. DL-based breast cancer analysis and detection have gained significant attention in the medical field due to their potential to improve accuracy, speed up diagnosis, and aid in early detection. DL algorithms, particularly CNNs, have shown promise in analyzing medical images, including mammograms, ultrasound scans, and histopathology images.

AI and ML have proven to be highly effective tools in the detection and treatment of various life-threatening diseases. These techniques have played a pivotal role in early diagnosis and treatment, significantly improving patients’ chances of survival. DL has been specifically designed to analyze critical factors that impact the detection and treatment of severe illnesses. One such example is the detection of breast cancer, which can be approached through genetic analysis or histopathological imaging. Genetic-level analysis can be costly, making histopathological imaging the more prevalent method for breast cancer detection. In this research, the authors have conducted a comprehensive systematic review of previous studies related to the detection and treatment of breast cancer using either genetic sequencing or histopathological imaging, with the assistance of DL and ML techniques [10]. This review aimed to provide valuable insights into the state of the field, highlighting the advancements and challenges encountered in these two approaches and the authors have provided recommendations and future research directions.

DL-based breast cancer analysis and detection covers a wide range of research studies and developments in the field [11]. This study explores the fusion of clinical parameters and thermal data using DL models for breast cancer diagnosis. The authors show how combining multiple sources of information can improve diagnostic accuracy. 

Furthermore, in addressing the limitation of inadequate training data, Generative Adversarial Networks (GANs) have gained prominence as image augmentation techniques. Thuy and Hoang [12] employed StyleGAN and conditional GAN (Pix2Pix) to generate synthetic datasets, known as the fake BreakHis datasets, derived from the original BreakHis dataset. 

The authors of [13,14] proposed a DL-based approach using transfer learning to classify breast cancer histopathological images. Their results demonstrate the effectiveness of pre-trained models for accurate classification. The thesis [15] applied transfer learning using ResNet18, Inception-V3Net, and ShuffleNet to perform binary and multiclass classification of breast cancer from histopathological images. Transfer learning offers faster and simpler training compared to training networks from scratch. The study used the BreakHis dataset, achieving high accuracy: 97.11% for ResNet18 in binary classification, and 94.17% for ResNet18 in multiclass classification, with other models also performing well [15]. 

Cruz-Roa et al. [16] surveyed and provided an overview of the use of DL and other ML techniques in analyzing breast cancer histopathology images. It covered various aspects, including feature extraction, classification, and tissue segmentation. Artificial Neural Network (ANN) techniques are frequently employed in the segmentation and classification tasks of breast histopathology images to increase the objectivity and accuracy of Breast histopathology Image Analysis (BHIA). The writers of the review [16] have provided a thorough synopsis of ANN-based BHIA approaches. For a more thorough analysis, they first divided the BHIA systems into deep and conventional neural networks. Next, pertinent research utilizing BHIA systems was showcased for histopathological examination.

The authors of [17] presented a CNN model for the classification of breast cancer histopathology images, emphasizing the application of transfer learning techniques. The CNNs were employed to categorize histopathological images of breast cancer from the publicly available BreaKHis dataset. The training process involved extracting image patches to train the CNN, and the amalgamation of these patches contributed to the final classification. The primary objective was to utilize high-resolution histopathological images from BreaKHis as input for existing CNN architectures, thereby avoiding the need for intricate and computationally demanding model adaptations.

Comparative analysis revealed that the CNN’s performance surpassed previously reported results obtained by alternative machine learning models that were trained with manually crafted textural descriptors. Notably, the CNN’s efficacy was demonstrated without necessitating complex architectural adjustments. Additionally, the authors explored the use of various pre-trained models to enhance classification accuracy. The study culminated in the fusion of different CNNs through simple rules, leading to an improvement in recognition rates. The evaluation process involved assessing the effectiveness of different pre-trained models to achieve superior classification accuracy [17].

A CNN model was used for the classification of histopathology images related to breast cancer, with a specific emphasis on utilizing transfer learning techniques. CNNs classified the breast cancer histopathological images from BreaKHis, a publicly available dataset. To train the CNN and determine the final classification, CNNs extracted picture patches. By using the high-resolution histopathological pictures from BreaKHis as input to the current CNN, this approach attempted to prevent model changes that could result in an architecture that is more intricate and computationally expensive. When compared to earlier published outcomes from other machine learning models trained with manually created textural descriptors, the CNN performance is superior. Eventually, some improvement in recognition rates was achieved by combining several CNNs using straightforward fusion methods. They assess various pre-trained models to increase classification accuracy [17].

With a similar amount of 1145 normal, benign, and malignant images, the authors of this study [18] employed four mammography imaging datasets with different deep CNN (Inception V4, ResNet-164, VGG-11, and DenseNet121) models as base classifiers. The Gompertz function is utilized by the authors to create fuzzy rankings of the base classification methods, and the decision scores of the base models are then adaptively blended to create the final predictions. This is an ensemble approach. In terms of accuracy and prediction, the fuzzy ensemble techniques fared better than the individual transfer learning methodologies and some sophisticated ensemble strategies (Weighted Average, Sugeno Integral). With a 99.32% accuracy rate, the Inception V4 ensemble model employs a fuzzy rank based Gompertz function. We anticipate the recommended strategy will be extremely helpful to medical professionals in detecting breast cancer cases early on, maybe resulting in an early diagnosis.

The timely and precise detection of breast cancer is pivotal in enhancing prognosis and elevating the patient survival rate to 50% [19]. Deep learning-based Computer-Aided Diagnosis (CAD) has demonstrated notable efficacy in the early diagnosis of breast cancer. With a focus on deep learning architectures used in breast cancer diagnosis, this review provides an extensive and analytical analysis of several important aspects, including model architecture for breast cancer diagnosis, datasets and image pre-processing, imaging techniques, performance metrics, and future research directions.

To identify and classify breast cancer, a novel deep learning method combining random forest classifier and deep convolutional neural networks is suggested [20]. The Random Forest Classifier improves the classifier precision while the Deep Convolutional Neural Network’s AlexNet model handles feature extraction. The pictures are taken from different mammography pictures that belong to pre-established datasets. When compared to state-of-the-art schemes, the proposed scheme performs better, according to the performance data. 

A comprehensive perspective [21] was adopted to establish a more optimal foundation for cultivating a thorough comprehension of Deep Learning (DL). Specifically, this review strove to offer a more exhaustive exploration of the crucial facets of DL, encompassing recent advancements in the field. The study employed Convolutional Neural Network (CNN) architectures, highlighting key features such as the AlexNet network and High-Resolution network. The authors addressed challenges and proposed solutions to elucidate prevailing research gaps, followed by an enumeration of major DL applications. Additionally, the research delves into computational tools, including FPGA, GPU, and CPU, elucidating their impact on DL approaches.

The authors of [22] thoroughly investigated the manifold applications of AI in breast cancer care, emphasizing both its revolutionary potential and current challenges. In the context of diagnosing and treating breast cancer, the focus is on AI applications in pathologic diagnosis, biomarker evaluation, and prognostic predictions pertaining to genetic changes, response to treatment, and prognosis. The study emphasized the importance of targeted outcomes to fully grasp the overall benefits that AI may bring to patient care, as well as the transformational potential of AI in the management of breast cancer. 

Ahn et.al. [23] performed a review of state-of-the-art DL techniques for breast cancer detection and diagnosis. They also discussed advancements and challenges in the field. These research studies represented a subset of the extensive research on DL-based breast cancer analysis and detection. Medical imaging uses Deep Neural Network (DNN) algorithms for segmentation, classification, and BC detection [23]. It also included a thorough examination of the imaging modalities used to identify BC, as well as an emphasis on their operation, advantages, and disadvantages. Finding the best imaging modalities and deep learning techniques to handle the massive information and produce accurate predictions was the main goal. The authors disclosed that pre-processing techniques like data augmentation, scaling, and normalization have been employed in several studies to lessen variability and overfitting in breast cancer pictures. Deep and shallow neural network designs are also applied to the analysis of BC pictures [24]. 

Using three pre-trained networks—VGG-16, VGG-19, and ResNet50—the authors of [25] showed the efficacy of transfer learning when compared to a fully trained network on a histopathological imaging modality. They have also examined the behavior of these networks for the purpose of classifying breast cancer independently of magnification. Simultaneously, they looked at how the amount of the training and testing data affected the networks under consideration. The best performance was achieved with a fine-tuned pre-trained VGG-16 with logistic regression classifier, which produced a 90–10% training–testing data splitting with 92.60% accuracy, 95.65% area under the ROC curve, and 95.95% accuracy precision score.

DL-based breast cancer analysis and detection have the potential to enhance the accuracy and efficiency of breast cancer diagnosis, ultimately improving patient outcomes and reducing healthcare costs [19]. However, translating these technologies into clinical practice requires collaboration among DL/ML experts, medical professionals, and regulatory bodies to ensure their safe and effective use in healthcare settings. In recent years, transfer learning has continued to evolve as a critical technique in improving the accuracy and efficiency of breast cancer detection and analysis. Researchers have explored various DL architectures, data augmentation strategies, and domain adaptation techniques to further enhance the performance of these models. Keeping up with the latest research in this dynamic field is essential for staying at the forefront of breast cancer diagnosis and analysis using transfer learning techniques. A comprehensive review on transfer learning-based breast cancer detection and analysis would encompass a range of studies and approaches that leverage pre-trained models and knowledge from related domains to improve the accuracy and efficiency of breast cancer diagnosis and analysis [26,27]. 

The suitable action for treating cancer is early detection; yet radiologists’ breast cancer screening is highly costly and time-consuming [28]. More significantly, there is a significant false-positive rate in traditional image analysis techniques for breast cancer. The essential characteristics influencing the diagnosis and management of breast cancer are extracted and analyzed using several breast cancer imaging modalities. Mammograms, ultrasounds, magnetic resonance imaging, histopathological pictures, and any combination of these imaging modalities can be subdivided into subgroups. Pathologists or radiologists examine pictures. The authors also looked at recent research that used a variety of breast imaging modalities and AI to diagnose breast cancer. Furthermore, they showcased the datasets that are currently accessible for breast cancer imaging modalities, which are crucial for creating AI-based algorithms. 

A fundamental investigation delved into the fundamentals of transfer learning and its diverse applications within the realm of image classification. Although not explicitly focused on breast cancer, the survey provides a crucial theoretical foundation for grasping the core principles of transfer learning in the context of medical image analysis. The authors conducted a thorough review of deep transfer learning, offering valuable insights into its broader application in image classification [29].

Since deeper neural networks are more challenging to train, the authors of [30] proposed a residual learning framework to facilitate the training of networks that are significantly deeper. Rather than learning unreferenced functions, the authors of this work reconstructed the layers as learning residual functions with reference to the layer inputs. Based on the empirical evaluation, it is known that these residual networks may be optimized more easily and that a significant increase in depth can improve accuracy. The scientists assessed residual nets on the ImageNet dataset that reach up to 152 layers, which is 8× deeper than VGG nets with a lower level of complexity. An ensemble of these residual nets presented an analysis of CIFAR-10 with 100 and 1000 layers, and they achieved an error of 3.57% on the ImageNet test set. On the COCO object detection dataset, the depth of representations showed a 28% relative improvement, which is crucial for many visual recognition applications. 

The researchers used deep learning in their work [31] to create an algorithm intended for the automated identification of diabetic macular edema and diabetic retinopathy in retinal fundus photos. With the help of a deep convolutional neural network—a type of neural network specifically designed for image classification—the model was trained using a retrospective dataset that included 128,175 retinal pictures. A panel of 54 US-licensed ophthalmologists and senior residents in ophthalmology carefully rated these photographs. The performance of the algorithm was then confirmed using two different datasets, each graded by at least seven board-certified ophthalmologists in the United States, guaranteeing a high level of intragrader consistency. The algorithm’s effectiveness, sensitivity, and specificity were evaluated to identify referable diabetic retinopathy, which included both referable diabetic macular edema and moderate-to-severe diabetic retinopathy. The panel of ophthalmologists’ majority opinion served as the basis for the reference standard. Two distinct operational points were selected from the development set to evaluate the algorithm: one was tuned for high specificity, and the other for high sensitivity [30].

Comparing the cascaded Deep Forest ensemble model [32] to other options, such as traditional DNNs and general ensemble learning techniques, the model achieved competitive classification accuracy. This is particularly noteworthy when working with training datasets that are unbalanced. It achieved this by using a cascade of decision trees to learn hyper-representations. Using multi-omics datasets and different configurations, a cascade Deep Forest model was employed in this study to classify breast cancer subtypes, notably IntClust and Pam50. The outcomes show an accuracy of 77.55% for ten subtypes and 83.45% for five subtypes. The cascaded Deep Forest classifier can achieve comparable accuracy to other techniques while delivering superior computational performance by utilizing gene expression data. The computation time recorded for classifying 10 subtypes is approximately 5 s, and for 5 subtypes, it is approximately 7 s [32]. 

In this paper [33], the research employed transfer learning and deep feature extraction techniques, leveraging pre-trained CNN models to tailor them to the specific problem under investigation. The investigation involved the utilization of AlexNet and VGG-16 models for extracting features, with subsequent fine-tuning performed specifically using AlexNet. The acquired features are subjected to classification using support vector machines (SVM). Thorough experimentation is conducted on a publicly accessible dataset of histopathologic breast cancer images, and accuracy scores are computed for evaluating performance. The results of the evaluation demonstrate that transfer learning outperforms deep feature extraction coupled with SVM classification in producing superior results [34].

In the research conducted by Sathiyabhama et al., a LeNet convolutional neural network (CNN) model was specifically crafted for the analysis of breast cancer data [35]. This model showed impressive accuracy in differentiating between benign and malignant tumors and successfully extracted discriminative features. By utilizing a corrected Rectified Linear Unit (ReLU) activation function, which is a modification of the traditional ReLU, the “dying ReLU” issue was resolved, and the extracted features’ discriminative ability inside the LeNet architecture was much increased. This adaptation resulted in improved performance for breast cancer data analysis, contributing to more precise and reliable detection and diagnosis of breast cancer, ultimately leading to enhanced patient outcomes. It played a crucial role in improving performance and training stability by mitigating the effects of internal covariate shift, addressing overfitting and reduce running time during the training process. A thorough evaluation of the developed classifier’s performance against benchmarking deep learning models revealed a higher recognition rate. The suggested LeNet model with corrected ReLU activation and batch normalization is effective in advancing breast cancer detection and diagnosis, as evidenced by the remarkable 89.91% accuracy in breast image identification [35].

Detecting the pectoral muscle is a crucial task that enhances the diagnostic accuracy of breast cancer detection. Early identification of the pectoral muscles plays a vital role in minimizing ambiguity between tumor cells and the pectoral muscle. Suppression of the pectoral muscle is essential for achieving accurate segmentation. Pectoral muscle identification is done using Otsu’s threshold, and connected constituent labeling is used to identify and remove connected pixels outside the breast region (classes 10 and 100, respectively) [36].

In the context of aiding pathologists in cancer analysis, CAD is introduced for the automated identification and diagnosis of breast cancer [37]. The CAD system in this study is built upon deep CNNs. Deep Learning (DL) signified a noteworthy progression in the field of artificial intelligence (AI), with notable accomplishments in image processing. On the other hand, convolutional neural networks, or ConvNets, usually required many images for training. To overcome this difficulty, features are extracted from a pre-trained network of the ImageNet challenge (ILSVRC) using transfer learning. Five well-known ConvNets, namely ResNet, VGG-19, VGG-16, Xception, and MobileNet, are utilized for feature extraction. This method helps reduce the computational load required for training ConvNets, limiting the hardware resources needed. ConvNets coupled with machine learning (ML) techniques are recognized for their superior performance compared to hand-crafted features in histopathology image analysis. Experimental results indicate that VGG-16, combined with the SVM approach, outperforms other configurations in automated histopathological image classification [37]. This underscores the effectiveness of leveraging pre-trained ConvNets and ML techniques for improved performance in histopathological image analysis and classification.

Breast cancer is the most widely reported maternal disease in the world, even though most instances are discovered much later in life. However, not all general hospitals regularly have mammography available for the investigation of breast cancer. The chance of breast cancer spreading may increase with a longer time between diagnosis and therapy. A computerized technique based on machine learning (ML) was developed to expedite the diagnosis of breast cancer and reduce the death rate. Improving the accuracy of ML algorithms for breast cancer diagnosis was the aim of this study. The classification and prediction of cancer as benign or malignant will be possible with the application of machine-learning techniques. Using the feature extraction technique of linear discriminant analysis (LDA) and the machine learning algorithms of random forest (RF) and SVM, this study examines the Wisconsin Breast Cancer Dataset. Accuracy results for the SVM with LDA and RF with LDA were 95.6% and 96.4%, respectively. Results from this study demonstrate the importance of improved prediction and how ML techniques can help. When compared to previous research, the findings of this study have validated the use of feature extraction for breast cancer prediction [38].

Diabetic Macular Edema (DME), a rare eye condition prevalent among diabetic patients, results from fluid accumulation in the macular region of the retina. Optical Coherence Tomography (OCT) has significantly improved the visualization of retinal structures. Despite being an advanced imaging modality, manual detection of DME in OCT images by medical professionals has been the norm. Leveraging deep learning algorithms with multiple layers has enhanced efficiency, enabling earlier DME detection. The authors have introduced a Lesion-based CNN (LCNN) algorithm designed for effective lesion detection, contributing to improved predictive capabilities. Upon conducting a comparative analysis with alternative deep learning models such as ResNet, VGG-16, and Inception, it was observed that the LCNN model surpasses them, exhibiting superior accuracy in detecting Diabetic Macular Edema (DME) [39].

In skin-based anomaly prediction, automatic region identification is fiercely competitive. DL methods are pivotal for this, yet most lack uncertainty quantification, leading to overconfidence and sample deviation. Addressing this, Monte-Carlo (MC) dropout was applied with the ResNet 50 DL model on the International Skin Image Collaboration (ISIC) Archive dataset. Effectively overcoming vanishing gradients and showcasing resilience to noise, this model excelled with previously unseen data. The MC dropout technique adeptly identified out-of-domain data, reducing training time. Consequently, the ResNet50 model achieved an impressive 89% accuracy in skin cancer classification [40].

The researchers [41] introduced a model called PSO CNN, which stands for Particle Swarm Optimization-based Convolutional Neural Network. This model was specifically developed to identify and classify retinopathy (DR) in color fundus images. The PSO CNN model comprises three components: preprocessing, feature extraction and classification.

In the initial preprocessing stage, unwanted noise from the input images is removed. Subsequently, the feature extraction process employs the PSO-CNN model to identify a valuable subset of features. In the end the extracted features are fed into a decision tree model to classify DR images. The PSO CNN model was tested using a DR database and the results of the experiment demonstrated its superiority compared to other methods that were compared. The outcomes suggest that the PSO-CNN model outperforms other approaches in the effective detection and classification of diabetic retinopathy from color fundus images [41].

Numerous comprehensive surveys have been conducted recently on the broader topic of deep transfer learning, both in general contexts and specifically concerning specialized tasks like image classification. Recognizing the significance of consolidating current knowledge and analyzing overarching patterns, our survey aims to formally define deep transfer learning and its application to image classification. We systematically assess the current state of the field, highlighting recent advancements and identifying existing gaps in knowledge. Our goal is to contribute insights that guide future progress, offering suggestions on how to address these gaps and propel the field forward. In our review, we particularly focus on deep transfer learning for image classification, emphasizing its relevance and potential. We present a detailed examination of where recent breakthroughs have occurred and pinpoint areas where further research is needed to advance our understanding. To bridge these knowledge gaps, we propose a transfer learning framework tailored for breast histopathology image classification. Through our literature survey, we not only outline the existing challenges but also offer insights into how transfer learning can be harnessed more effectively in the context of breast histopathology. Recognizing the necessity for automated methods to analyze diverse breast screening images and support radiologists in image interpretation, we advocate for the application of novel approaches. Our work aligns with the growing trend of leveraging AI, particularly deep learning algorithms, to enhance the early detection and treatment of various cancers, with a specific focus on breast cancer. The combination of AI methods and the wide range of datasets from imaging techniques offer a unique chance to surpass the constraints of current approaches in analyzing breast cancer. This ultimately can lead to outcomes and increased survival rates.

There is reason to be hopeful that breast cancer can be successfully treated if it is detected and addressed. That is why many researchers have developed automated methods using learning to accurately predict the growth of cancer cells based on imaging techniques. It has been consistently proven that the effective approach for classifying images involves training deep models with a significant amount of labeled data. However, in real-world situations it is often difficult to obtain the amount of training data to achieve performance. In these cases, transfer learning can be employed to enhance performance.

### 2.1. Research Contributions 

The concatenated transfer learning for classifying the breast histopathology images is listed as follows:

i. Enhanced Diagnostic Capabilities: They contribute to the improvement of diagnostic capabilities for breast cancer by demonstrating the effectiveness of the proposed three-stage concatenated model. They also provide evidence of how the combined use of deep learning and transfer learning can significantly enhance the accuracy of histopathological data analysis.
ii. Personalized Treatment Planning Support: this highlights the potential impact of the proposed model in aiding personalized treatment planning for breast cancer patients. It showcases how the model’s accurate classification of cancer subtypes or stages can inform more tailored and effective treatment strategies.
iii. Rigorous Validation Techniques: These contribute to the field by employing rigorous validation techniques to ensure the accuracy and reliability of the proposed model. They also share insights into the validation methods used and their role in establishing the credibility of the model’s performance.

### 2.2. Objectives

The research objectives of classifying the breast histopathology images using transfer learning are listed as follows:

i. To evaluate Feature Extraction Performance

Perform a comprehensive examination of the feature extraction stage to evaluate its effectiveness in utilizing DNN for capturing pertinent information from histopathological images. Explore the specific tumor morphology and microenvironment features that are successfully extracted, contributing significantly to the subsequent stages of the analysis.

ii. Assess Transfer Learning Impact

Evaluate the effectiveness of transfer learning in fine-tuning the extracted features, focusing on how knowledge from unrelated domains enhances the model’s ability to discern subtle patterns in breast cancer tissue samples. Investigate the transferability of knowledge and identify the optimal pre-trained models for the given task.

iii. Optimize Classification Network

Fine-tune the classification network to ensure accurate categorization of breast cancer samples into different subtypes or stages. Explore variations in network architectures and hyperparameters to enhance the classification performance.

iv. Generalization and Robustness

Investigate the generalization capabilities of the model by evaluating its performance on diverse datasets and different institutions. Assess the robustness of the model against variations in image quality and staining techniques commonly encountered in real-world histopathological data.

v. Comparative Analysis

Conduct a comparative analysis with existing state-of-the-art methods to showcase the superiority of the proposed concatenated model in breast cancer histopathology analysis.

These research objectives and contributions aim to provide a comprehensive understanding of the proposed model’s strengths, limitations, and potential impact on advancing breast cancer diagnosis and treatment planning.

## 3. Proposed Transfer Learning Based Novel Concatenated Classifier

In the context of breast cancer histopathological analysis, transfer learning can be particularly beneficial when working with medical images, such as mammograms or histopathological slides. By starting with a pre-trained model and fine-tuning it on a specific breast cancer dataset, we can harness the power of DL while addressing the challenges posed by limited data availability and the need for specialized expertise in medical image analysis. This paper presents a novel approach for breast histopathology image classification using Concatenated Transfer Learning. The proposed architecture iteratively refines the model’s performance through multiple stages, leveraging the knowledge transferred from the previous stage while introducing specific adaptations for binary classification. The detailed image classification process has several steps which include model creation, layer freezing, compilation, and the construction of a concatenated model list, highlighting its application for breast histopathology image analysis. Breast histopathology image classification plays a pivotal role in the early diagnosis and prognosis of breast cancer and our research work here is a Transfer Learning-based Concatenated approach to further enhance the classification accuracy. Concatenating the traditional DL models, VGG-16 and MobileNet, ResNet and DenseNet have been proven to be effective in this task.

Concatenated Transfer learning is appropriate in analyzing breast cancer data for several reasons:

1. Limited Data Availability: Collecting a large and diverse dataset for breast cancer analysis can be challenging. Transfer learning allows you to leverage pre-existing datasets that have been used for related tasks, such as image classification, and adapt them for breast cancer detection tasks. This helps mitigate the data scarcity issue.
2. Feature Extraction: Pre-trained models in transfer learning have already learned useful features from vast amounts of data. These features can be valuable for identifying patterns and characteristics in breast cancer images, reducing the need for handcrafted feature engineering.
3. Improved Generalization: Transfer learning enables models to generalize better from one domain (e.g., general image recognition) to another (e.g., breast cancer detection). By fine-tuning a pre-trained model on breast cancer data, you can make it more specialized and accurate for the target task.
4. Reduced Training Time and Resources: Training DL models from scratch require substantial computational resources and time. Transfer learning starts with a pre-trained model and fine-tune it on the specific dataset, which typically requires less time and resources compared to training from scratch.
5. Better Performance: Transfer learning often results in models that perform better than models trained solely on limited domain-specific data. The pre-trained model has already learned valuable representations that can be fine-tuned to achieve high accuracy in breast cancer detection.
6. Interpretability: Transfer learning can help improve the interpretability of your model. By leveraging features learned from a larger dataset, the model may capture meaningful patterns that are easier to understand and interpret, aiding in medical diagnosis.
7. Continuous Learning: As more data becomes available, you can continually update and fine-tune your model to improve its performance. Transfer learning allows you to adapt your model to changing circumstances and data distributions.

### 3.1. Design of Transfer Learning Based Concatenated Classifier

**Figure 2:**
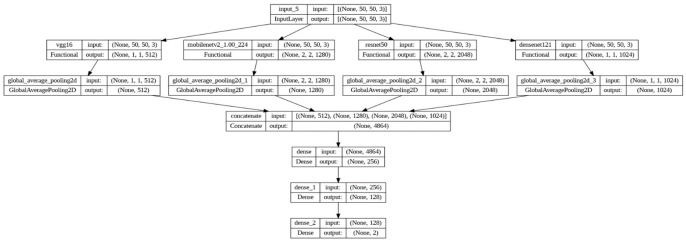
Classifier with concatenated information across four levels of layers.

**Figure 3:**
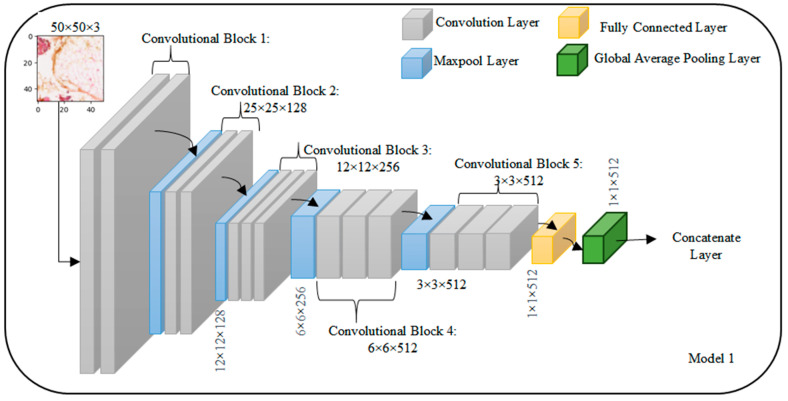
VGG-16.

**Figure 4:**
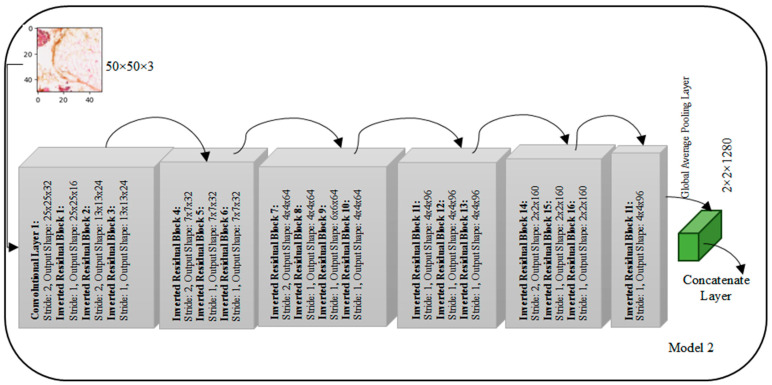
MobileNetV2.

**Figure 5:**
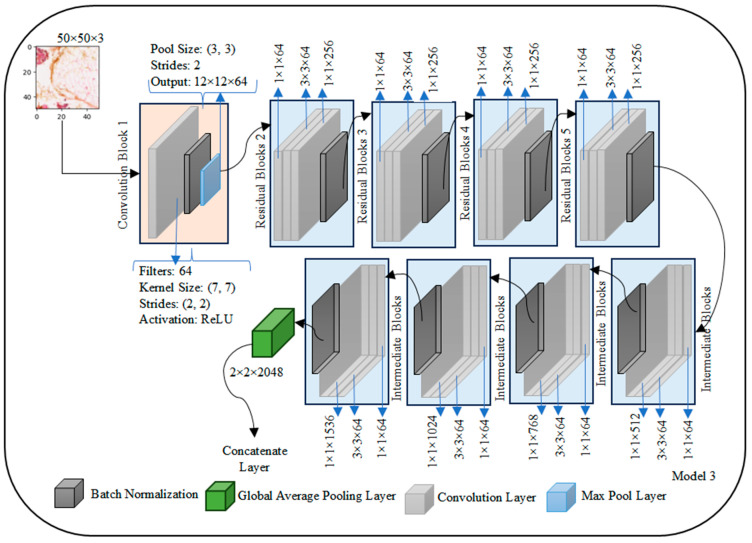
ResNet50.

**Figure 6:**
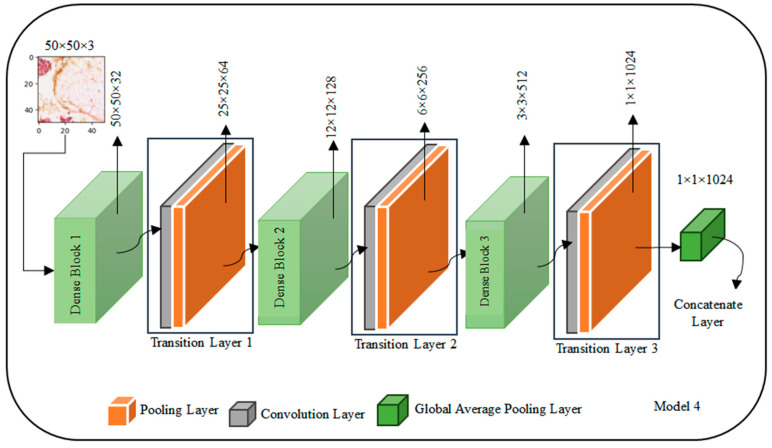
DenseNet121.

**Figure 7:**
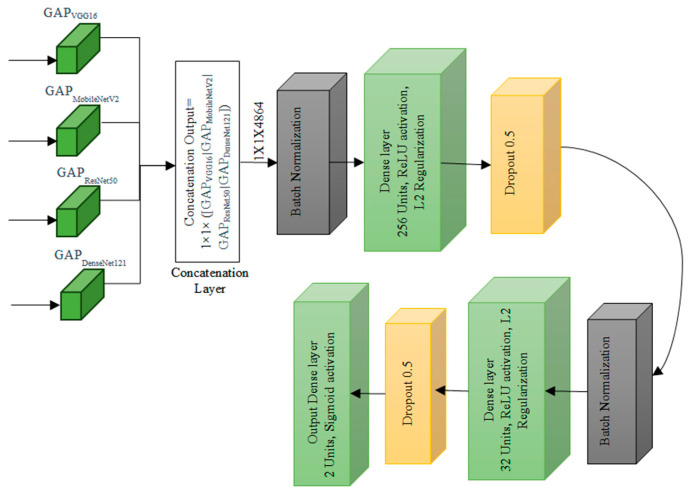
Concatenated Model Architecture.

The Transfer Learning-based Concatenated model for classifying breast histopathology images is comprehensively described in this section. In this research work, a novel concatenated classifier with three levels is designed for breast histopathology image classification. The input images have dimensions of 50 × 50 × 3. The model architecture and training process are illustrated in the following (Figure 2, Figure 3, Figure 4, Figure 5, Figure 6 and Figure 7).

To apply transfer learning effectively in a concatenated setup in analyzing breast cancer data, the following steps are to be performed:

1. Data Preparation: Annotate and clean histopathology breast cancer dataset thoroughly, ensuring accurate labeling. Split the dataset into training, validation, and test sets and the common split ratio is 70, 15 and 15, respectively.
2. Input Layer: mathematically, the input layer can be represented as X, where X is the input tensor with dimensions (batch_size, 50, 50, 3) (batch_size, 50, 50, 3).
3. Pre-trained Models: Select pre-trained DL models that have shown success in related tasks. Models like VGG-16, MobileNetv2, ResNet50 and DenseNet121 are popular choices for image-related tasks which also matched the complexity of the histopathological dataset.

### 3.2. VGG-16 Architecture

The VGG-16 architecture is structured with multiple convolutional blocks which are shown in Figure 3, each comprising convolutional layers followed by ReLU activation functions, and interspersed with pooling layers for spatial dimension reduction. The input layer accepts images with a shape of 50 × 50 × 3. In the first convolutional block, the initial layer has 64 filters, resulting in an output shape of 50 × 50 × 64. The subsequent convolutional layer maintains the same filter count and output shape, followed by a pooling layer with an output shape of 25 × 25 × 64. The pattern is repeated in the following blocks: the second block has two convolutional layers with 128 filters and a pooling layer reducing the output to 12 × 12 × 128. The third and fourth blocks follow a similar structure with 256 and 512 filters, respectively. The fifth block consists of three convolutional layers with 512 filters each, and a final pooling layer reduces the output to 1 × 1 × 512. The architecture concludes with a Global Average Pooling layer, yielding a final output shape of 1 × 1 × 512. This standardized VGG-16 configuration, with its progressively increasing filter counts and spatial dimension reduction through convolution and pooling, culminates in a global average pooling layer for effective feature aggregation before the final classification.

### 3.3. MobileNet Architecture

The MobileNetV2 architecture, depicted in Figure 4, adopts a unique structure comprising multiple convolutional blocks, each housing inverted residual blocks with varying filter counts. The input layer expects images with a shape of 50 × 50 × 3. In Block 1, the first convolutional layer, with an expansion factor, employs 32 filters with a stride of 2, resulting in an output shape of 25 × 25 × 32. Subsequent inverted residual blocks follow, featuring filter counts of 16, 24, and 24 with different stride values, modifying the output shapes accordingly. Block 2 continues with three inverted residual blocks employing 32 filters each, while Block 3 introduces four inverted residual blocks with filter counts of 64. Block 4 features three inverted residual blocks with 96 filters each. The subsequent blocks, Block 5 and Block 6, consist of three and one inverted residual blocks, respectively, with filter counts of 160 and 320. The architecture concludes with a Global Average Pooling layer, yielding a final output shape of 1 × 1 × 1280, deviating from the earlier mentioned 2 × 2 × 1280. This MobileNetV2 design, characterized by inverted residual blocks and varying filter configurations across blocks, is tailored for efficient and effective feature extraction in image processing tasks.

### 3.4. ResNet 50 Architecture

The ResNet50 architecture is structured to efficiently process images with an initial input layer expecting a shape of (50, 50, 3) representing height, width, and RGB channels, respectively which is shown in Figure 5. The first convolutional block consists of a 7 × 7 convolutional layer with 64 filters and a ReLU activation function, resulting in an approximate output shape of (24, 24, 64). Batch normalization is applied, followed by a max-pooling layer with a pool size of (3, 3) and strides of (2, 2), yielding an approximate output shape of (12, 12, 64). The subsequent architecture includes residual blocks (2 to 5), each containing residual units with shortcut connections. A representative residual unit in Block 2 involves three convolutional layers with appropriate filter sizes, kernel sizes, and activation functions, culminating in element-wise addition with the shortcut connection to maintain the same output shape.

Intermediate blocks (6 to 9) follow a similar structure to residual blocks, gradually increasing the number of filters. The architecture then employs a global average pooling layer, resulting in an approximate output shape of (1, 1, 2048), which condenses the spatial dimensions of the previous layer into a single value for each feature map. The final output layer is a fully connected (dense) layer with two units for binary classification and a sigmoid activation function.

In summary, ResNet50 exhibits a total parameter count of around 25 million, which may vary based on specific configurations and weight initialization. The model’s complexity is notable due to its depth and inclusion of skip connections, enhancing its capability to capture intricate features. It is worth noting that the actual output shapes may vary slightly depending on specific configurations and padding choices in the individual layers.

### 3.5. DenseNet121 Architecture

The DenseNet architecture is presented in Figure 6 with illustrative values for a 50 × 50 × 3 input image, exemplifies its unique structure involving dense blocks and transition layers. Starting with the input image of shape (50, 50, 3), the first dense block, denoted as Dense Block 1, processes the input, and produces an output shape of (50, 50, C_out1), using hypothetical values such as 64 channels. Following this, Transition Layer 1 transforms the output to (25, 25, C_out2), with, for instance, 128 channels. The second dense block, Dense Block 2, operates on this transformed output, resulting in an output shape of (25, 25, C_out3) with hypothetical values like 256 channels. Transition Layer 2 further modifies the output to (12, 12, C_out4), with 512 channels as an illustrative example. The process continues with Dense Block 3 and Transition Layer 3, ultimately producing an output shape of (6, 6, C_out6) with 128 channels.

Following the dense blocks and transition layers, a Global Average Pooling Layer is applied to the (6, 6, C_out6) input, resulting in an output shape of (1, 1, C_out6), where C_out6 is a hypothetical value, such as 64 channels. The architecture concludes with a Fully Connected Layer, functioning as the output layer for classification. It takes the (1, 1, C_out6) input and generates a final output shape of (1, 1, Num_classes), where Num_classes represent the number of classes, illustrated here with 10 hypothetical classes. It is important to note that the values C_out1 through C_out6 used here are for explanatory purposes and may vary in practice based on the specific DenseNet variant chosen and the hyperparameters defined during the model design process. The selection of growth rates and other configuration choices contributes to the variability in these channel counts, adapting the architecture to the specific requirements of the given task.

### 3.6. Concatenated Model Architecture

Concatenation Layer concatenates the output tensors from global average pooling of each pre-trained model. Mathematically, if *X_vgg_*, *X_mobile_*, *X_resnet_*, *X_dense_* are the outputs from each pre-trained model, the concatenated output *X_concat_* is given by:

$$X_{concat}=Concatenate(\left[X_{vgg}X_{mobile}X_{resnet}X_{dense}\right]$$

Freeze the top 4 classification layers of VGG-16 and MobileNetV2 models, top 3 layers of ResNet50 and top 4 layers of DenseNet121 are considered which are specific to the original task, which is a generic process of classification normally done in any pretrained models and is depicted in Figure 6. The fifth level represents the integration of knowledge from the four levels, combining the outputs from all the four levels with the original breast histopathology image data. The number of layers is determined based on the different combinations of layers that were experimented and the best ones are chosen.

By building a series of concatenated models with varying learning rates and progressively freezing layers, the system leverages the power of pre-trained models VGG-16, MobileNetV2, ResNet50, and DenseNet121 to refine their performance over multiple stages. This method not only enhances the model’s accuracy but also facilitates knowledge transfer from one stage to the next, making it highly efficient for classifying breast histopathology images. Incorporating this approach into breast cancer diagnosis can potentially lead to more accurate and reliable results, contributing to improved patient care and outcomes.

Global Average Pooling Layer for each Pre-trained Model: The global average pooling operation reduces spatial dimensions to 1 × 1 by taking the average of all values in each channel. For each pre-trained model, the operation can be represented as

$$GAP\left(X\right)=\frac{1}{H\times W}\sum_{i=1}^{H}\sum_{j=1}^{W}X_{ij}$$

where *H* and *W* are the height and width of the feature map, and *X_ij_* represents the value at position (*i*, *j*) in the feature map. In the first level of the concatenated model, the focus is on feature extraction from input images. This stage comprises a series of convolutional layers, each playing a crucial role in capturing different aspects of the images. 

Batch Normalization Layer 1 normalizes the input by adjusting and scaling the activations. Mathematically, for a batch *B* and a feature *x* in *X_concat_*, the batch normalization operation can be represented as

$$BatchNorm\left(x\right)=\frac{x-\left(\mu_{B}\right)}{\sigma_{B}+\epsilon }\times \gamma +\beta$$

where *μ_B_* and *σ_B_* are the mean and standard deviation of the batch, *γ* and *β* are learnable parameters, and *ϵ* is a small constant for numerical stability.

Dense Layer 1 is the first dense layer which applied a linear transformation to the input. Mathematically, if *W*_1_ is the weight matrix, *b*_1_ is the bias vector, and *ReLU* is the rectified linear unit activation function, the operation can be represented as:

$${\mathrm{Dense}}_{1}(X)=ReLU(X\cdot W_{1}+b_{1})$$

It begins with 256 units, each having a 1 × 1 dimension, followed by a *ReLU* activation and L2 regularisation. 

Then, Dropout Layer 1 is added to dropout randomly sets a fraction of input units to zero during training to prevent overfitting while preserving valuable information. Mathematically, the operation can be represented as

$${Dropout}_{1}(X)=dropout\_mask⊙X$$

where ⊙ represents element-wise multiplication and *dropout_mask* is a binary mask with probabilities of dropping out, and to enhance generalization, a dropout rate of 0.5 is introduced here. 

Batch Normalization Layer 2 is applied after the first dropout layer. The second dense layer is like the first dense layer which has 32 units. Mathematically, if *W*_2_ is the weight matrix, *b*_2_ is the bias vector, and *ReLU* is the activation function and L2 regularisation is used, the operation can be represented as

$${\mathrm{Dense}}_{2}(X)=ReLU(X\cdot W_{2}+b_{2})$$

Dropout Layer 2 is applied after the second dense layer with a dropout rate of 0.5. The output layer produces the final classification probabilities. Mathematically, if *W_out_* is the weight matrix and bout is the vector, and *σ* is the sigmoid activation function, the operation can be represented as

$$\mathrm{Output}(X)=\sigma (X\cdot W_{\mathrm{out}}+b_{\mathrm{out}})$$

The combined data is flattened and directed through a dense layer that consists of 2 units. A Sigmoid activation function is applied to this layer is introduced to improve model robustness. The ultimate output from this stage is a binary classification, determining whether an image is benign or malignant, based on the knowledge and insights integrated from the previous levels. These mathematical representations provide an overview of the operations performed at each layer in the concatenated model. The specific values for parameters like weights, biases, and dropout rates are learned during the training process. 

The proposed concatenated Transfer Learning offered a systematic and effective way to improve the classification of breast histopathology images. The model is compiled using the Adam optimizer, binary cross entropy loss, and accuracy as the metric. This method not only enhances the model’s accuracy but also facilitates knowledge transfer from one stage to the next, making it highly efficient for classifying breast histopathology images. Incorporating this approach into breast cancer diagnosis can potentially lead to more accurate and reliable results, contributing to improved patient care and outcomes.

## 4. Experimental Evaluation

### 4.1. Dataset Description

The “Breast Ultrasound Images Dataset” is available at Kaggle [42]. Breast cancer can develop at any different part of the breast. The most common form of breast cancer is Invasive Ductal Carcinoma (IDC). IDC can be detected through various methods such as mammography, ultrasound, biopsy and so on. Through biopsy, histopathological images are derived. The dataset used for training and testing for the image classification model was the Breast Histopathology Images dataset. This dataset consisted of a total of 277,524 patches of images sized 50 × 50, which were broken down from 162 whole mount images. Within these patches, there were 198,738 IDC-negative and 78,786 IDC-positive. There were 279 patients, and each file contained images of IDC-positive and -negative patients. Each data in the dataset was formatted into a list type, which consisted of the Image Path and its class 0 or 1, 0 being IDC-negative and 1 being IDC-positive.

### 4.2. Details of Tools Used

We conducted our experiments using Google Colab, a cloud-based Jupyter notebook environment. To access the image sources, we connected Google Colab to Google Drive. The model training process was greatly accelerated by the presence of GPUs, in Google Colab specifically configured with type A100 and 80 GB RAM. For building learning models, we utilized the user neural networks API called Keras 2.6.0, which operates on top of TensorFlow 2.7.0 a powerful open-source machine learning framework. The seamless integration of Keras and TensorFlow, with Google Colab allowed us to effortlessly import the libraries and run code within an environment. This integration not simplified model development but also maximized GPU resource utilization. Python 3.10.11 was used for implementation purposes.

### 4.3. Model Evaluation

The evaluation of the model’s performance involves assessing metrics, including accuracy, precision, recall, F1 score and the area, under the operating characteristic curve (AUC ROC). To implement a concatenated transfer learning model, we have chosen four successful and reliable models VGG-16, MobilnetV2, Resnet50 and DenseNet121 Convolutional Neural Network architectures for a classification task. These models are showing better performance in classifying huge number of images and eventually the Concatenated transfer learning involves training multiple stages of a model where each stage builds upon the knowledge transferred from the previous stage and produced extraordinary performance which is presented in Table 1.

#### 4.3.1. Stages of Training

The concatenate consists of multiple stages, each represented by a DL model. The number of stages is determined by the length of concatenated models, which is initialized earlier. Each stage is trained sequentially, with each stage building on the knowledge from the previous one. The current stage’s model is trained using the fit method with training and validation data. Steps_per_epoch and validation_steps are calculated based on the batch size to determine how many batches to process per epoch. The number of training epochs is specified by epochs_per_stage[stage], which can be different for each stage. During training, the code collects the following metrics for each stage:

- Training accuracy
- Validation accuracy
- Training loss
- Validation loss

These metrics are appended to lists (train_accs, val_accs, train_losses, and val_losses) for later analysis. After training a stage, the trainable status of all previous stages is set back to False. This ensures that earlier stages do not continue to fine-tune when training subsequent stages.

#### 4.3.2. Fine-Tuning Previous Stages

If it’s not the first stage (stage > 0), earlier stages in the concatenate are fine-tuned while keeping the layers of the current stage frozen. This is accomplished by setting the trainable attribute of earlier stage models to True and recompiling the model with a specific learning rate for the current stage. Fine-tuning earlier stages allows the model to adapt to the task while retaining knowledge from later stages.

#### 4.3.3. Model Evaluation on Test Data

After training each stage, the model’s performance is evaluated on a separate test dataset using the evaluate method. The test accuracy is printed to assess how well the model generalizes to unseen data. The code implements a concatenate transfer learning approach for binary classification. It trains multiple stages of models progressively, allowing each stage to adapt to the task while retaining knowledge from the previous stages. This approach can be beneficial when working with limited data or when fine-tuning is required for a specific task. The collected metrics help monitor the performance of each stage and assess the overall effectiveness of the concatenate.

The model consists of three stages, each of which is a sequential model. The first stage model is initialized with the pre-trained VGG-16 model, with the top classification layers excluded. The second and third stage models are initialized with the output of the previous stage model. Each stage model consists of a Flatten layer, a Dense layer with the specified number of neurons and the ReLU activation function, a Dropout layer with a rate of 0.5, and an output layer with the specified number of classes and the sigmoid activation function. All layers in the base_model is frozen except for the last convolutional block in the stage model. This is to prevent the pre-trained features from being overwritten during training.

The training of stage models follows a sequential process, commencing with the initial stage model and progressing through to the ultimate stage model. At each stage, the model undergoes training using the output from the preceding stage model. Once all four stages are trained, the outputs from these models are concatenated, and the classification process is reiterated based on a meticulously chosen and fine-tuned layer configuration to generate the final predictions of the model. Employing progressive training in a concatenated transfer learning model proves highly effective in enhancing the performance of deep learning models, especially in tasks characterized by limited data or complexity. It is essential to acknowledge that training in a concatenated transfer learning model may necessitate more time compared to a single model, given that each stage in the concatenated model requires individual trainin. 

## 5. Results and Discussion

Histopathology images are analyzed using concatenated DL models for several reasons:

1. Complexity of histopathological images: Histopathological images are highly complex and contain intricate details. They often feature various structures, textures, and patterns that are crucial for accurate disease diagnosis. A concatenated DL model can capture both low-level and high-level features in a hierarchical manner, allowing it to better understand the intricate details within these images. The concatenate structure can filter out noise in the initial layers and focus on more complex patterns in subsequent layers.
2. Large variability in tissue types: Histopathological images encompass a wide range of tissue types, each with unique characteristics. A single DL model may struggle to capture the heterogeneity of these tissues. A concatenated model can be designed to handle this diversity by employing different classifiers at each stage, each specialized for specific tissue types or disease states.
3. Reduction of false positives and negatives: In medical applications, especially in histopathology, false positives (misclassifying healthy tissue as diseased) and false negatives (missing diseased tissue) can have serious consequences. Concatenated models can mitigate this by employing a primary classifier that filters out clear negatives and leaves more challenging cases for further scrutiny by a secondary classifier, improving both precision and recall.
4. Transfer learning benefits: Concatenated models often incorporate transfer learning, using pre-trained models for feature extraction. This is particularly advantageous in histopathological image analysis due to the limited size of medical datasets. Transfer learning allows the model to leverage knowledge gained from large and diverse image datasets, adapting it to the specific histopathology task.
5. Robustness and generalization: The concatenated structure allows for greater robustness and generalization to different datasets. The primary classifier focuses on general features, while the secondary classifier can be fine-tuned to adapt to dataset-specific nuances. This ensures that the model can perform well on unseen data.
6. Improved interpretability: The concatenated model’s multi-stage structure can also improve the interpretability of the results. By examining the decisions made at each stage, pathologists and researchers can gain insights into why a particular classification was made, which is crucial for trust and acceptance in medical applications.
7. Incremental learning and adaptation: Concatenated models can be designed to adapt over time, enabling incremental learning as more data becomes available. This is particularly valuable in medical settings where new insights and data are continually emerging.
8. Faster convergence: The scheduled learning rate allows the model to converge faster during the initial phases of training, saving computational resources.
9. Enhanced model performance: The resulting model exhibits superior performance in breast cancer histopathological data analysis, with higher accuracy, sensitivity, and specificity and is presented in Table 2.

**Figure 8:**
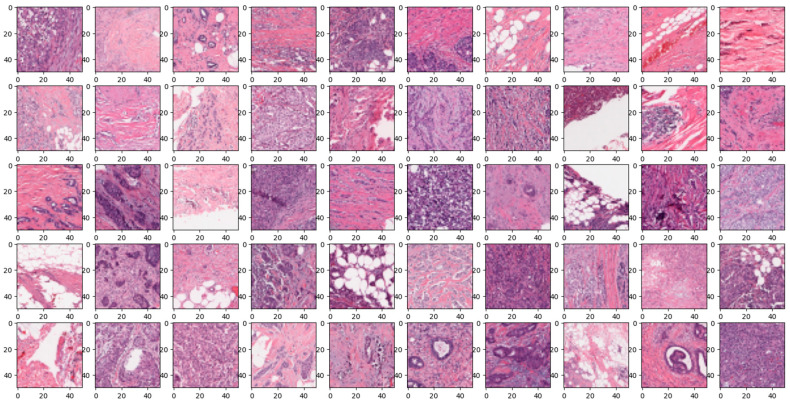
Sample true images (50 × 50).

**Figure 9:**
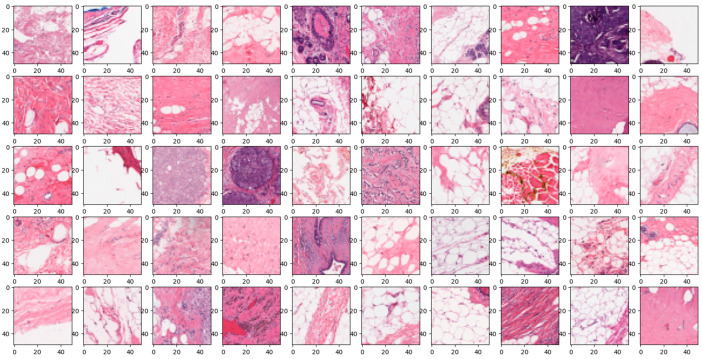
Sample false images (50 × 50).

In the realm of breast histopathology image classification, the analysis is facilitated through concatenated DL models. This approach is particularly well-suited to manage the intricate and diverse nature of the data, mitigating the occurrence of false positives and negatives. Leveraging transfer learning, these models enhance robustness, generalization, and interpretability, making them conducive to incremental learning. The application of concatenated models proves highly effective for tasks such as disease diagnosis and tissue classification in histopathology. This not only holds the potential to refine patient care but also promises improved clinical outcomes. A total of 277,524 images were utilized in this study. A sample true and false images are presented from the input image dataset in Figure 8 and Figure 9.

The confusion matrix shown in Table 2 reveals key metrics: True Positive (29,719), False Positive (919), False Negative (329), and True Negative (10,660). Row entries in the table contribute to the determination of Positive Predictive Values (a) and Negative Predictive Values (b), while column entries aid in establishing Sensitivity (c) and Specificity (d). The Positive Predictive Value and Negative Predictive Value are crucial metrics in evaluating the performance of a classification model, especially in the context of breast histopathology image analysis. Positive Predictive Value represents the probability that instances predicted as positive by the model are truly positive, offering insights into the reliability of positive predictions. Negative Predictive Value, on the other hand, signifies the probability that instances predicted as negative are indeed negative, providing an assessment of the model’s ability to accurately identify true negatives.

False negatives in this context mean that the model failed to classify 329 instances as positive when, in reality, they were positive cases. In breast histopathology image analysis, this might correspond to instances where the model missed identifying cancerous tissues or abnormalities. In medical settings, minimizing false negatives is crucial to ensuring that patients with actual health issues receive timely attention. The comparison of model performance to real-life practice would involve assessing whether the model’s false negative rate aligns with acceptable levels (<0.5% of false negatives) in clinical settings and whether the model provides value in aiding healthcare professionals rather than replacing their expertise. To reduce the number of false positives, the threshold for positive predictions can be lowered, then the model can become more sensitive, potentially capturing more positive cases. However, this might come at the cost of an increase in false positives. Continuous improvement of feature selection and engineering may enhance the model’s ability to identify subtle patterns associated with positive cases. In the medical field, these values play a pivotal role for practitioners in tailoring treatments to patients. A high Positive Predictive Value ensures that when the model identifies a condition as positive, it is likely accurate, reducing the chances of unnecessary treatments or interventions. This is particularly crucial in breast histopathology, where precision in identifying abnormalities is essential for determining the course of treatment. Similarly, a high Negative Predictive Value assures practitioners that when the model predicts a negative result, it is likely correct. This is essential for ruling out conditions accurately, preventing unnecessary stress, and averting potentially invasive procedures when they are not warranted.

Furthermore, Sensitivity measures the ability of the model to correctly identify positive instances, while Specificity gauges its ability to accurately identify negative instances. In the context of breast histopathology, high Sensitivity ensures that the model is effective in detecting true positive cases, which is crucial for identifying potential health risks early. High Specificity ensures that the model is adept at avoiding false positives, reducing unnecessary concern and interventions. In summary, the careful consideration of these metrics in breast histopathology image analysis empowers medical practitioners with a comprehensive understanding of the model’s performance. This, in turn, aids in making informed decisions regarding patient treatments, minimizing the risk of unnecessary procedures, and ultimately enhancing the quality of patient care.

**Figure 10:**
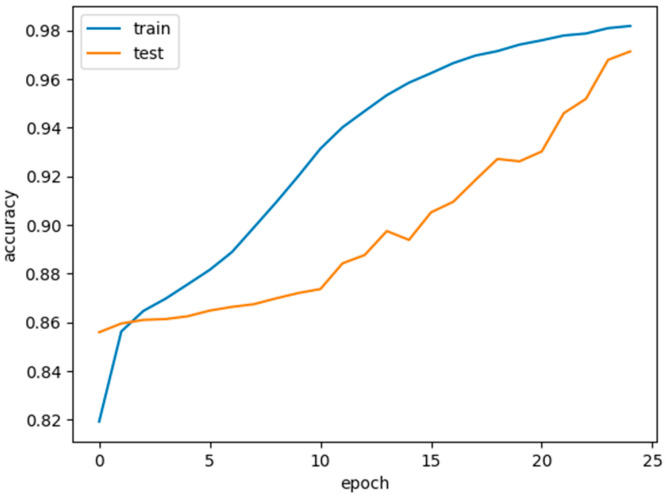
Training and Testing Accuracies of Concatenated Model.

**Figure 11:**
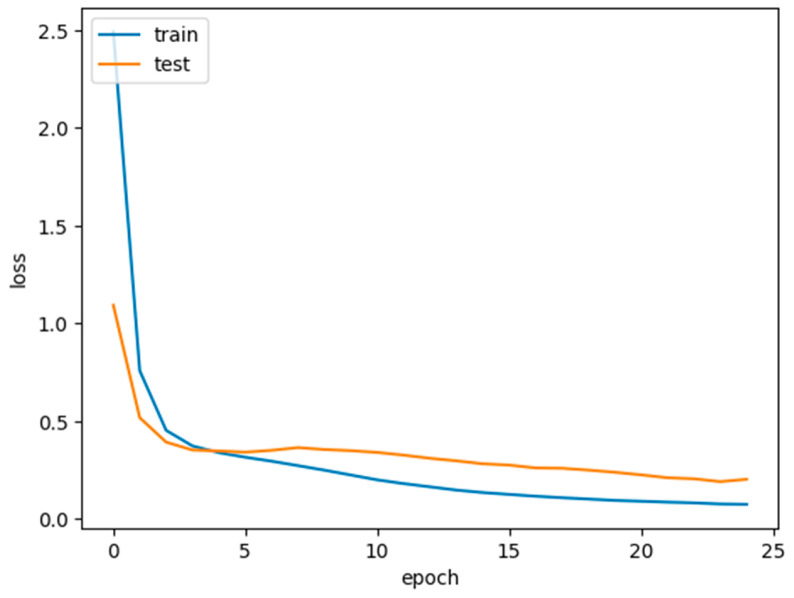
Concatenated model loss.

The concatenated model demonstrates impressive results, achieving a training accuracy of 98% and a testing accuracy of 96.8%. The training and testing losses initiate at 2.5 and 1.1, respectively, during the first epoch and gracefully degrade as the epochs progress, reaching 25 runs which are presented in Figure 10 and Figure 11. The inference from these results can be analyzed in terms of both training and testing aspects:

1. Training accuracy and loss: high training accuracy (98%): A high training accuracy indicates that the model has effectively learned patterns and features from the training data. However, an excessively high training accuracy may suggest overfitting, where the model becomes too tailored to the training data and may not generalize well to new, unseen data. Decreasing Training Loss: The decreasing training loss during epochs implies that the model is improving in minimizing errors on the training data. A gradual decrease indicates effective learning and convergence.
2. Testing accuracy and loss: high testing accuracy (96.8%): A high testing accuracy suggests that the model performs well on unseen data, demonstrating its ability to generalize beyond the training set. This is a positive indication of the model’s robustness.

Graceful degradation of testing loss: The fact that the testing loss degrades gracefully over epochs suggests that the model is not overfitting the testing data. A steady decline in testing loss indicates that the model is adapting well to the testing set without showing signs of overfitting.

The gradual degradation of training and testing losses suggests that the model is learning steadily without exhibiting signs of overfitting. This is favorable for the model’s ability to generalize to new, unseen data. The choice of concatenated models, leveraging transfer learning, and the careful tuning of hyperparameters contribute to the model’s ability to handle the complexity of histopathological data and achieve superior performance.

In summary, the concatenated model not only exhibits excellent performance on the training and testing sets but also demonstrates robustness and generalization capabilities, making it a promising approach for histopathological image classification. The impact of the high training and testing accuracies indicate that the concatenated model is adapting well to the testing set without showing signs of overfitting. A steady decline in testing loss indicates that the model generalizes well to new data.

**Figure 12:**
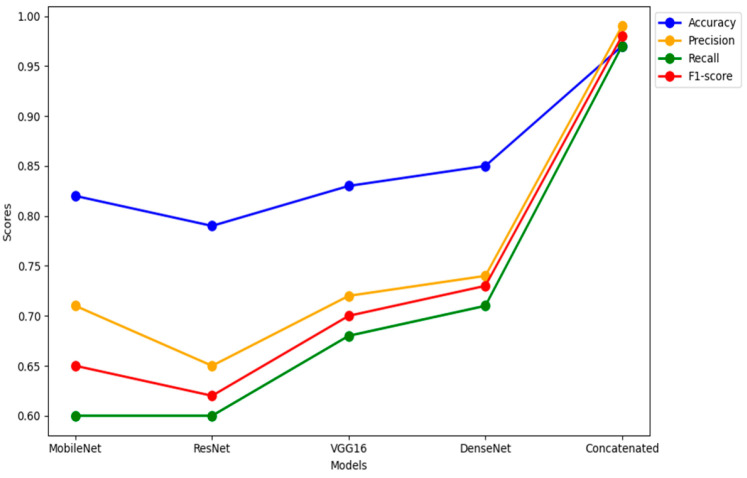
Performance of the concatenated model.

Table 1 and Figure 12 provides performance metrics for different models based on accuracy, precision, recall, and F1-score and interpretation of the metrics are as follows:

1. Accuracy: The Concatenated Model outperforms individual models in terms of accuracy, achieving a remarkable accuracy of 97%. This indicates that the concatenated model is effective in making correct predictions on the overall dataset.
2. Precision: Precision measures the accuracy of positive predictions. The Concatenated Model has a precision of 99%, indicating that when it predicts a positive class, it is highly likely to be correct. It excels in minimizing false positives.
3. Recall: Recall (Sensitivity) measures the ability of the model to capture all positive instances. The Concatenated Model achieves a recall of 97%, suggesting it effectively identifies a large proportion of the actual positive instances.
4. F1-Score: F1-Score is the harmonic mean of precision and recall. The Concatenated Model achieves a high F1-Score of 98%, indicating a balance between precision and recall. This suggests a strong overall performance.

Comparative analysis: Among the individual models, DenseNet121 has the highest accuracy, precision, recall, and F1-Score, indicating its superior performance compared to VGG-16, MobileNetV2, and ResNet50. The Concatenated Model significantly outperforms all individual models in accuracy, precision, recall, and F1-Score. This emphasizes the effectiveness of the concatenated approach in improving model performance.

Implications:

The Concatenated Model demonstrates a high level of accuracy and precision, making it a robust choice for applications where minimizing false positives is crucial, such as medical diagnostics. The high recall suggests that the Concatenated Model is effective in capturing a large proportion of positive instances, which is critical in scenarios where identifying all positive cases is essential.

The strong F1-Score reinforces the balanced performance of the Concatenated Model, indicating its suitability for applications where precision and recall are equally important.

In summary, the Concatenated Model stands out as a powerful approach, combining the strengths of pretrained architectures and transfer learning to achieve superior performance in comparison to individual models.

**Figure 13:**
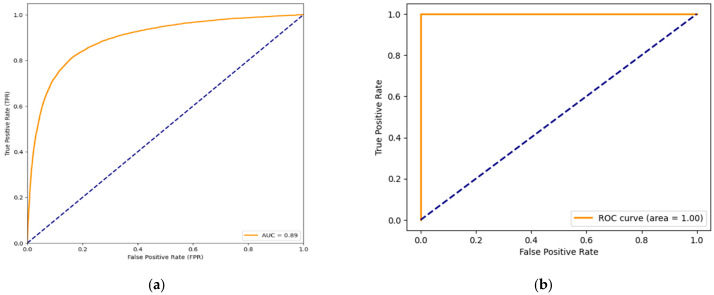
(**a**,**b**): RoC curves for the concatenated model.

The concatenated model’s Receiver Operating Characteristic (ROC) curve value of 0.89, along with occasional instances of a perfect ROC curve (1) are presented in Figure 13a,b, carry several implications:

1. High accuracy and ROC curve: The models training accuracy of 98% and testing accuracy of 96.8% indicate that it has learned effectively from the training data and can apply its knowledge to data. The ROC curve visually demonstrates how well the model distinguishes between false positive instances. With an ROC curve value of 0.89 the model is considered to have an ability to differentiate between negative cases.
2. Perfect ROC curve (1) in some runs: A perfect ROC curve with a value of 1 implies that the model achieved perfect discrimination between positive and negative instances. In repeated runs, consistently achieving a perfect ROC curve is noteworthy and suggests a high level of model stability and reliability.

The implications of an ROC curve value of 0.89 indicates a high discrimination ability of the model in distinguishing between true positive and false positive instances. This is particularly important in medical applications where accurate classification is crucial. Achieving a perfect ROC curve in repeated runs signifies that the model’s performance is consistent and robust across multiple executions. This is a positive indicator of the model’s reliability and reproducibility. The high testing accuracy coupled with a strong ROC curve suggests that the Concatenated Model generalizes well to new, unseen data. This is essential for the model’s practical utility in real-world scenarios. While the training accuracy is high, the model’s ability to generalize is further supported by the ROC curve results. The occasional perfect ROC curve indicates that the model does not overfit the training data and maintains strong performance on diverse datasets. ROC curves provide insights into the model’s performance across different decision thresholds. The optimal threshold can be selected based on specific requirements, balancing sensitivity, and specificity according to the application’s needs. From this, the Concatenated Model demonstrates robust performance, effective generalization, and consistent discrimination ability, as indicated by high accuracy and ROC curve values. These findings suggest that the model is well-suited for the classification task, especially in medical applications, where precision and reliability are of utmost importance.

Stringent quality control measures are imperative in the diagnostic process to ensure accuracy and reliability. Regular audits and adherence to established protocols play a vital role in this. Standardizing procedures and providing ongoing training for pathologists foster consistency, reducing errors. Integration of advanced technologies, such as digital pathology and AI, acts as a second layer of analysis, catching potential errors and improving diagnostic precision. Collaboration among specialists provides a comprehensive view of patient cases, minimizing the risk of overlooking vital information. Regular case reviews and peer consultations enhance the team’s knowledge, reducing diagnostic errors. Clinical information guides interpretations, preventing misdiagnoses. Effective communication, patient education, and involvement in shared decision-making are essential for managing expectations. The dynamic field of histopathology continuously advances, contributing to enhanced diagnostic accuracy.

The model enhances diagnostic accuracy and efficiency for histopathologists without replacing their expertise. Integration into daily hospital use involves seamless incorporation into existing workflows, ensuring easy access for pathologists analyzing breast cancer samples. Implementation includes rigorous validation studies, regulatory approvals, and clinical guidelines. The technology aids subtype classification, identifying molecular subtypes for personalized treatment strategies, and distinguishing in situ from invasive breast cancer. Continuous improvement and collaboration with healthcare professionals ensure the model’s relevance, reliability, and alignment with clinical needs for enhanced patient care.

## 6. Conclusions

Our study demonstrated the profound impact of the Transfer Learning-based concatenated model in advancing the accuracy and efficiency of breast cancer histopathological data analysis. The integration of well-established pretrained models, including VGG-16, MobileNetV2, ResNet50, and DenseNet121, into a concatenated architecture showcases a remarkable training accuracy of 98%. This signifies a significant improvement over traditional methodologies, highlighting the potential of deep learning techniques in revolutionizing breast cancer diagnostics and treatment planning. The systematic benchmarking against individual classifiers on histopathological data consistently reveals the superiority of the concatenated model, emphasizing its efficacy in leveraging the strengths of multiple pretrained architectures. This model not only enhances diagnostic precision but also holds promise in contributing to more personalized and informed treatment strategies for individuals diagnosed with breast cancer. Further, efforts can be made to improve the interpretability of the concatenated model. Enhanced transparency in model decision-making can install greater confidence among healthcare practitioners and facilitate a seamless integration of the model into clinical practice. Collaborations with healthcare institutions and practitioners can facilitate the seamless integration of the model into diagnostic processes, ensuring its real-world utility. We aimed to propel the concatenated model towards practical implementation, ultimately contributing to improved patient outcomes and marking a significant stride in the intersection of artificial intelligence and healthcare.

## Figures and Tables

**Table 1:** Accuracy, Precision, Recall and F1-Score of DL Models.

Model	Accuracy	Precision	Recall	F1-Score
VGG-16	0.83	0.72	0.68	0.70
MobilenetV2	0.82	0.71	0.60	0.65
Resnet50	0.79	0.65	0.60	0.62
Densenet121	0.85	0.74	0.71	0.73
Concatenated Model	0.97	0.99	0.97	0.98

**Table 2:** Confusion Matrix.

Screening Test Results		
Has the Condition		Does Not Have the Condition
**Positive**	a	b
	True positive	False positive
	(29,719)	(919)
**Negative**	c	d
	False negative	True negative
	(329)	(10,660)

## Data Availability

Publicly available datasets were analyzed in this study. This data can be found here: http://gleason.case.edu/webdata/jpi-dl-tutorial/IDC_regular_ps50_idx5.zip (accessed on 15 May 2023).
